# Sustainable phytoremediation of saline soils using *Atriplex hortensis* L.: a case study from Bizerte Lagoon, Northern Tunisia

**DOI:** 10.3389/fpls.2025.1613594

**Published:** 2025-09-16

**Authors:** Salma Sai Kachout, Sana Dhane, Salah BenYoussef, Abderrazak Tlili, Molka Douili, Ferdaous Guesmi, Aziza Zoghlami

**Affiliations:** ^1^ Animal and Forage Productions Laboratory (LR16 INRAT 01), National Institute of Agricultural Research of Tunisia (INRAT), Carthage University, Tunis, Tunisia; ^2^ The National Agronomic Institute of Tunisia, Tunis, Tunisia; ^3^ Higher Institute of Cultural Sciences and Heritage Professions of Tunis, Tunis, Tunisia; ^4^ The Institute of Arid Regions of Médenine, Médenine, Tunisia

**Keywords:** soil salinity, halophyte, *Atriplex hortensis*, phytoremediation, forage crop, salt stress, Tunisia

## Abstract

Soil salinization is a growing global concern that undermines agricultural productivity and land sustainability. *Atriplex hortensis*, a C_3_ annual halophyte, has shown promise as both a forage crop and a phytoremediation agent in saline environments. This field study assessed the potential of *A. hortensis* for reclaiming salt-affected soils in the Bizerte Lagoon region of Northern Tunisia. Plants were cultivated under naturally saline field conditions, and their physiological, morphological, and growth responses were monitored over six months. Results showed that increasing salinity significantly reduced biomass, with shoot dry mass decreasing from 168 g to 73.2 g, and root dry mass from 25.9 g to 15.6 g. Salinity stress significantly reduced chlorophyll fluorescence (ΦPSII) from 0.54 to 0.41 and stomatal conductance (gs) from 0.45 to 0.28 mol m^-2^ s^-1^, while relative water content (RWC) remained stable above 88%. Leaf area declined by 54%, limiting photosynthetic surface, whereas specific leaf area remained unchanged, indicating preserved leaf tissue density and structural integrity in *Atriplex hortensis*. A notable reduction in soil electrical conductivity from 3.48 to 2.26 dS m^-1^ (−35%) was observed, indicating effective phytodesalination. Despite reduced biomass, *A. hortensis* maintained physiological stability and exhibited signs of salt tolerance. These findings support the use of *A. hortensis* as a dual-purpose species for forage production and soil desalination in arid and semi-arid ecosystems.

## 1 Introduction

Soil salinization is a critical environmental challenge that severely diminishes soil fertility and agricultural productivity, leading to significant economic losses. It results from both natural processes and anthropogenic activities, including poor irrigation management, excessive fertilizer use, and climate-induced shifts in water availability (FAO, 2016). Globally, salinity affects nearly 20% of irrigated lands approximately 45 million hectares posing a serious threat to food security and ecosystem integrity (Kumar and Sharma, 2020). Tackling this issue requires sustainable and innovative strategies to rehabilitate salt-affected soils while sustaining agricultural production.

Among the most promising solutions is the use of halophytes, which are naturally adapted to thrive in saline environments. Species within the Chenopodiaceae family are especially notable for their resilience to salinity and drought. Eu-halophytes, a subset of halophytic plants, can tolerate extreme salinity levels often surviving in sodium chloride concentrations exceeding 200 mM (up to 5%). According to the eHALOPH database, 333 eu-halophyte species are recorded across 70 plant families, with approximately 75% concentrated in just 19 families (Flowers and Colmer, 2015; Tiziana et al., 2022). Genera such as *Atriplex*, *Salicornia*, *Suaeda*, and *Salsola* have shown considerable potential for use in saline agriculture and ecological restoration (Qadir et al., 2007; Glenn et al., 1999). Many halophytes contribute to biological desalination by hyperaccumulating toxic ions such as sodium (Na^+^) and chloride (Cl^-^), and some tolerate heavy metal contamination (Manousaki and Kalogerakis, 2011; Timothy and Colmer, 2008). These traits make them ideal candidates for phytoremediation of saline and degraded lands. Moreover, halophytes are increasingly valued not only as models for studying salt tolerance but also as multipurpose crops for forage, biomass production, and land rehabilitation using saline or marginal water resources (Abdelly et al., 2006; Fedoroff et al., 2010; Shabala, 2011). Their ability to extract and store salts offers a cost-effective and environmentally friendly means to reduce soil salinity, particularly in areas where conventional remediation techniques are not feasible (Qadir et al., 2007, 2008). Field evidence supports their effectiveness: for example, *Sesuvium portulacastrum* can remove up to 1 ton of Na^+^ per hectare, while species like *Suaeda maritima* and *S. portulacastrum* have shown strong salt uptake capabilities in field applications (Rabhi et al., 2009, 2010a, 2010b; Ravindran et al., 2007). Several studies have identified *Atriplex hortensis* as a moderately salt-tolerant species, capable of surviving and growing in both saline and drought-affected environments. Research on seed germination and early seedling development demonstrates that *A. hortensis* can withstand moderate salinity levels, with germination rates gradually declining as salt concentration increases, yet maintaining viability under conditions that inhibit many non-halophytic species (Kaya et al., 2013; Niu et al., 2017). Its natural distribution frequently includes saline soils and disturbed habitats where water availability is variable, highlighting its notable resilience to drought stress (Ungar, 1991). Key ecophysiological mechanisms, including osmotic adjustment, ion regulation, and enhanced antioxidant enzyme activity, have been reported as contributors to its ability to sustain physiological functions under various abiotic stresses (Xu and Li, 2019). Together, these traits underscore the potential of *Atriplex hortensis* as a valuable candidate for phytoremediation and sustainable forage production in marginal and salt-affected soils.

The red cultivar of *A. hortensis* was selected in this study for its demonstrated salt extraction efficiency under field and greenhouse conditions (Jeschke and Stelter, 1983; Ungar, 1991; Sai Kachout et al., 2011; Morteau et al., 2009; Pan et al., 2016). Like many *Atriplex* species, it retains absorbed sodium and other ions within internal tissues, likely compartmentalizing them in vacuoles to mitigate ionic toxicity. This internal accumulation strategy contributes to soil desalination through biomass harvesting while allowing the plant to maintain cellular homeostasis under saline conditions (Flowers and Colmer, 2008). While some congeners, such as *A. canescens*, retain salts within their tissues and act more as internal accumulators (Litalien and Zeeb, 2020), *A. hortensis* exhibits an excretory strategy that facilitates both plant tolerance and soil desalination. The genus *Atriplex* is widely recognized for its exceptional tolerance to a range of environmental stressors, including drought, salinity, heavy metal contamination, and extreme temperatures (Benzarti et al., 2012; Manousaki and Kalogerakis, 2011; Shahbaz and Ashraf, 2013). These traits underscore its strong potential for use in the ecological restoration of degraded lands, particularly those impacted by saline irrigation, industrial effluents, or road runoff, where conventional crops fail to establish or persist.

This study aims to evaluate the phytoremediation potential and forage value of *Atriplex hortensis* under field conditions in Bizerte Lagoon, Northern Tunisia. By monitoring growth, biomass production, physiological traits, and changes in soil salinity, we assess the species’ capacity to adapt and improve soil health, contributing to sustainable agricultural development in salt-affected areas.

## 2 Materials and methods

### 2.1 Study area

**Figure 1:**
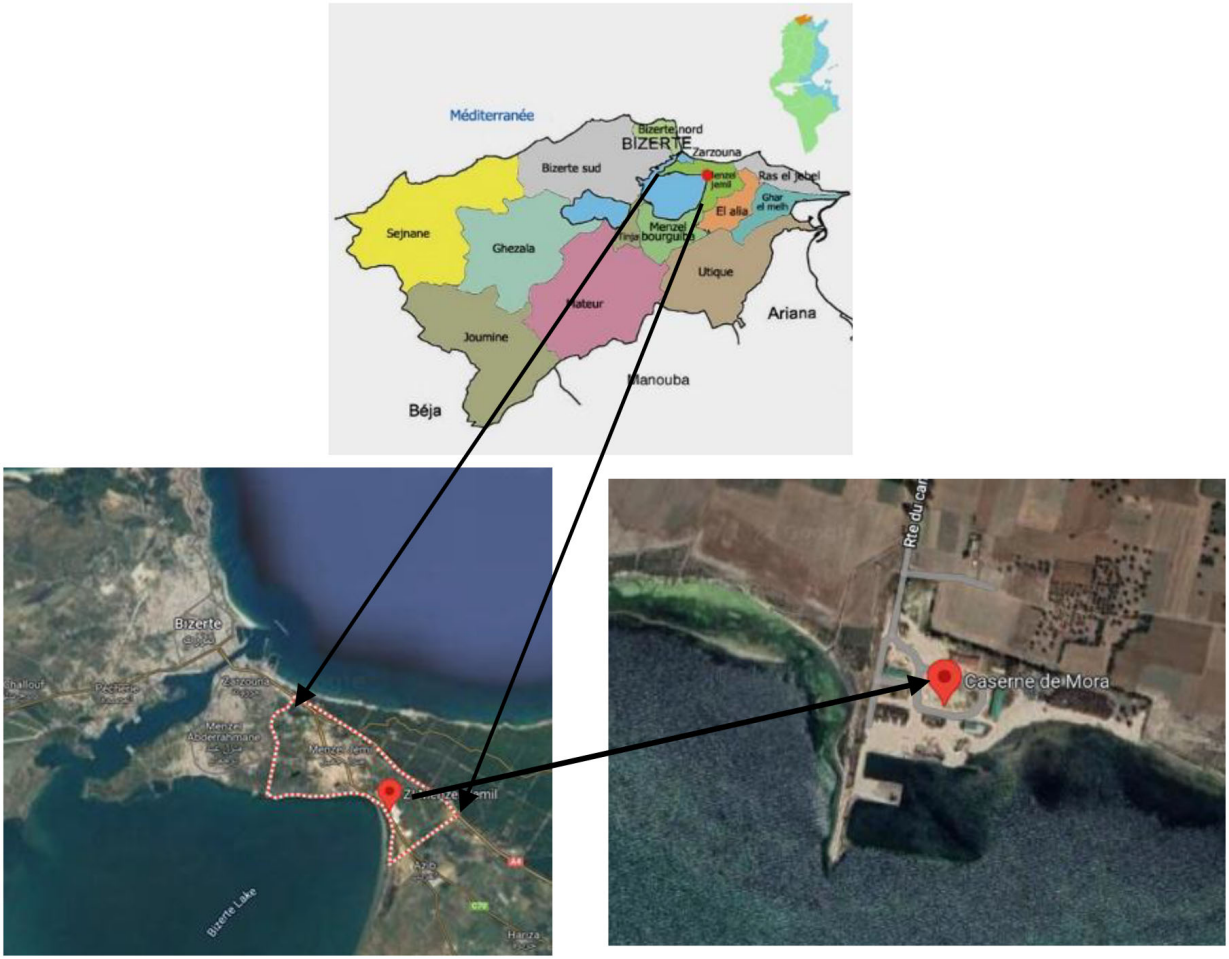
Study area location, Irrigated Perimeter Caserne de Mora, Menzel Jemil, Governorate of Bizerte, Tunisia.

The field trial was conducted during the 2022–2023 growing season at a naturally saline site located within the irrigated perimeter of Caserne de Mora, Menzel Jemil (37°24′ N, 9°91′ E; altitude: 26 m), in the governorate of Bizerte, Northern Tunisia (**Figure 1**). The region experiences a sub-humid Mediterranean climate with a mean annual rainfall ranging between 600 and 800 mm. Average monthly temperatures vary from 5 °C in winter (January) to 30°C in summer (August), with a distinct dry season typically extending from May to September.

The experimental site consisted of heterogeneous soils ranging from sandy to clay-silty textures. The land had a previous history of vegetable cultivation but was abandoned due to progressive salinization. To provide a reference for comparison, a nearby non-saline plot (~1 km away) with comparable soil texture and land use history but no documented salinity issues was selected as control. Both plots were evaluated for baseline soil properties prior to sowing, ensuring the absence of confounding variables such as irrigation quality, drainage, and cultivation practices.

### 2.2 Soil analysis

Prior to sowing, composite soil samples were collected from the topsoil layer (0–10 cm depth) of both the saline and control plots. Sampling was conducted using a stainless-steel auger at five random locations per plot. Samples were homogenized, air-dried at room temperature, and sieved to <2 mm to remove debris and coarse fragments.

The analyzed parameters included the electrical conductivity (EC) of the saturated paste extract, measured with a conductivity meter, as well as soil pH, determined in a 1:2.5 soil–water suspension using a calibrated pH meter. Additionally, exchangeable cations (Na^+^, K^+^, Ca²^+^, Mg²^+^) were extracted with 1 M ammonium acetate (pH 7.0) and quantified via atomic absorption spectrophotometry. Chloride content was also assessed, extracted from soil samples using deionized water and measured through argentometric titration with silver nitrate (AgNO_3_).

Plant samples were gently washed with distilled water prior to EC measurement to remove surface salts. The analytical procedures followed standard protocols as described by Jackson (1973) and Stanford and English (1949). Results indicated moderate to high salinity levels at the test site (EC = 3.48 dS m^-1^) and non-saline conditions at the control site (EC < 1 dS m^-1^). These values are consistent with FAO salinity classification thresholds and justified the selection of the two contrasting sites.

### 2.3 Plant material and experimental design

Seeds of *Atriplex hortensis* were collected from mature plants cultivated at the experimental station of Mornag (INRAT, Tunisia) during the 2021 growing season. Under Mediterranean conditions, the species typically begins flowering in March. The one-thousand seed mass (TSM) was approximately 7.9 g, based on the average of three independent seed samples. Prior to sowing, seeds were manually cleaned, and no chemical or physical pre-treatments were applied. Sowing was conducted under open field conditions in December 2022, using evenly spaced rows with standardized seeding depth and density to ensure uniform establishment across all plots.

A total of six harvests were carried out at regular 30-day intervals, corresponding to 30, 60, 90, 120, 150, and 180 days after sowing (DAS), covering the full vegetative growth cycle. At each time point, five plants per plot were randomly selected and harvested for biometric and physiological measurements.

The experimental layout followed a completely randomized design (CRD) with two main treatments (saline *vs*. non-saline control) and six sampling dates. Each treatment × time combination was replicated three times, with each replicate consisting of an individual plot. Data collected included shoot and root dry biomass, chlorophyll fluorescence (ΦPSII), stomatal conductance, relative water content, and post-harvest soil EC.

### 2.4 Biomass measurement

At each sampling point, plants were harvested and separated into leaves, stems, and roots. Tissues were oven-dried at 60 °C for 72 hours and weighed. Dry biomass accumulation (g plant^-1^) and shoot-to-root ratios were calculated. Soil around the root zone was carefully excavated, and roots were gently washed with water to remove adhering soil without damaging secondary roots.

Leaf area (LA, cm²) was measured using scanned images analyzed in ImageJ software. Specific leaf area (SLA, cm² g^-1^) was calculated as:


$$\text{SLA}=\text{Leaf Area}({\text{cm}}^{2})\times \text{Dry Leaf Mass}\;(\text{g})$$

### 2.5 Leaf gas exchange and chlorophyll fluorescence

Monthly measurements of leaf gas exchange and chlorophyll fluorescence were performed on the fully expanded third leaf from the apex of the main stem to ensure consistency across samples. For each treatment (saline and control), measurements were taken on five plants per plot, with three leaves per plant, resulting in 15 replicates per treatment. Light-adapted chlorophyll fluorescence (ΦPSII), Electron transport rate (ETR), Stomatal conductance (gs) and Vapor pressure deficit (VPD) were conducted between 10:00 a.m. and 12:00 p.m. under ambient field conditions using a LI-COR LI-600 Porometer/Fluorometer (LI-COR, NE, USA).

Chlorophyll fluorescence parameters were calculated as:

-
ΦPSII=Fm'−FsFm'\Phi_{PSII}

-
ETR=ΦPSII×PPFD×0.5×0.84ETR

Where PPFD is the photosynthetic photon flux density and the constants represent energy partitioning and leaf absorbance.

### 2.6 Plant water status

Leaf water potential (Ψ_h_) was determined using a Scholander-type pressure chamber (Model 600, PMS Instrument Company, Albany, OR, USA) in accordance with established protocols. Fully expanded and healthy leaves were carefully excised and immediately sealed in plastic bags to minimize water loss prior to measurement. All measurements were conducted during early morning hours to reduce diurnal variation. Relative water content (RWC) was determined via fresh weight (FW), turgid weight (TW), and dry mass (DM) using the formula:


RWC(%)=FW−DM/TW−DM×100

Where, FW was recorded immediately after sampling, TW after rehydration of leaves in distilled water for 24 hours at 4°C, and DM after oven-drying samples at 70°C for 48 hours until constant weight.

### 2.7 Statistical analysis

After confirming data normality (Shapiro-Wilk test), we conducted a two-way ANOVA to evaluate the effects of salinity treatment (control *vs*. saline), time (six growth stages), and their interaction. The time factor was treated as a fixed effect rather than repeated measures, due to the experimental design and data structure. Means were compared using Tukey’s HSD test at a significance level of p < 0.05. Pearson’s correlation coefficients (two-tailed) were calculated to assess relationships among morphological and physiological parameters. Additionally, heatmap clustering was performed to visualize the complex responses (morphological and physiological) under salinity stress allowing comprehensive changes assessment. All statistical tests were conducted using OriginPro 2022b software.

## 3 Results

### 3.1 Plant growth and biomass accumulation

Salinity significantly reduced the growth of *Atriplex hortensis*, with total dry biomass decreasing by 56% compared to control conditions (**Table 1**). Shoot dry mass dropped from 168 g to 73.2 g, while root dry mass declined from 25.9 g to 15.6 g, indicating a greater sensitivity of shoot tissues to salt stress. Biomass accumulation increased progressively from 30 to 180 days after sowing (DAS), peaking at 127.8 g in control plants. Under saline conditions, fresh biomass remained consistently lower across all stages, with the largest growth observed during the reproductive phase (120–180 DAS). Notably, the root-to-shoot ratio increased under salinity, suggesting an adaptive reallocation of resources toward root development to support water and nutrient acquisition. This response reflects a common strategy among salt-tolerant species, contributing to partial resilience in saline environments.

**Table 1:** Fresh and dry matter yield of leaves, stems and total biomass of *Atriplex hortensis* subjected to successive salinity-contaminated soil (mean ± SD, n = 5).

Month	Fresh matter (FM)			Dry matter (DM)		
	Leaf	Stem	Total	Leaf	Stem	Total
January	1.4 ± 0.2^d^	0.4 ± 0.1^e^	1.8 ± 0.3^d^	0.25 ± 0.04^c^	0.12 ± 0.01^e^	0.37 ± 0.1^e^
February	16.5 ± 0.5^c^	3.7 ± 0.2^d^	20.2 ± 0.7^d^	4.2 ± 0.1^b^	1.3 ± 0.1^d^	5.5 ± 0.2^d^
March	20.6 ± 0.8^b^	14.9 ± 0.5^c^	25.5 ± 1.3^d^	5.9 ± 0.2^b^	8.6 ± 0.5^c^	14.5 ± 0.7^c^
April	24.2 ± 0.7^a^	20.1 ± 0.7^c^	44.3 ± 1.4^c^	8.2 ± 0.4^a^	11.6 ± 0.6^c^	19.8 ± 1.0^c^
May	27.5 ± 0.9^a^	85.3 ± 2.2 ^b^	112.8 ± 3.1^b^	9.3 ± 0.7^a^	30.2 ± 1.5^b^	39.5 ± 2.2^b^
June	19.6 ± 0.6^b^	108.2 ± 4.1^a^	127.8 ± 4.7^a^	6.5 ± 0.9^b^	66.7 ± 3.4^a^	73.2 ± 4.3^a^

Means not followed by the same upper case letter are different (P<0.05).

Plant height increased progressively in both treatments, with consistently lower values under salinity. At 180 DAS, plants under control reached 114.7 ± 4.3 cm, while those under salinity attained only 89.6 ± 3.8 cm reflecting a 22% reduction (**Table 2**). This trend was observed throughout the growth cycle, indicating that salinity significantly affected vegetative development but did not fully inhibit growth.

**Table 2:** Mean plant height (cm) of *Atriplex hortensis* at successive growth stages under control and saline conditions (mean ± SD, n = 5).

DAS (Days after sowing)	Plant height	
	Control (cm)	Saline (cm)
30	12.4 ± 1.2^e^	9.1 ± 0.8^e^
60	35.6 ± 2.4^d^	27.8 ± 1.9^d^
90	61.3 ± 3.1^c^	46.7 ± 2.5^c^
120	89.2 ± 4.6^b^	67.4 ± 3.3^b^
150	106.5 ± 5.0^a^	82.1 ± 4.1^a^
180	114.7 ± 4.3^a^	89.6 ± 3.8^a^

**Figure 2:**
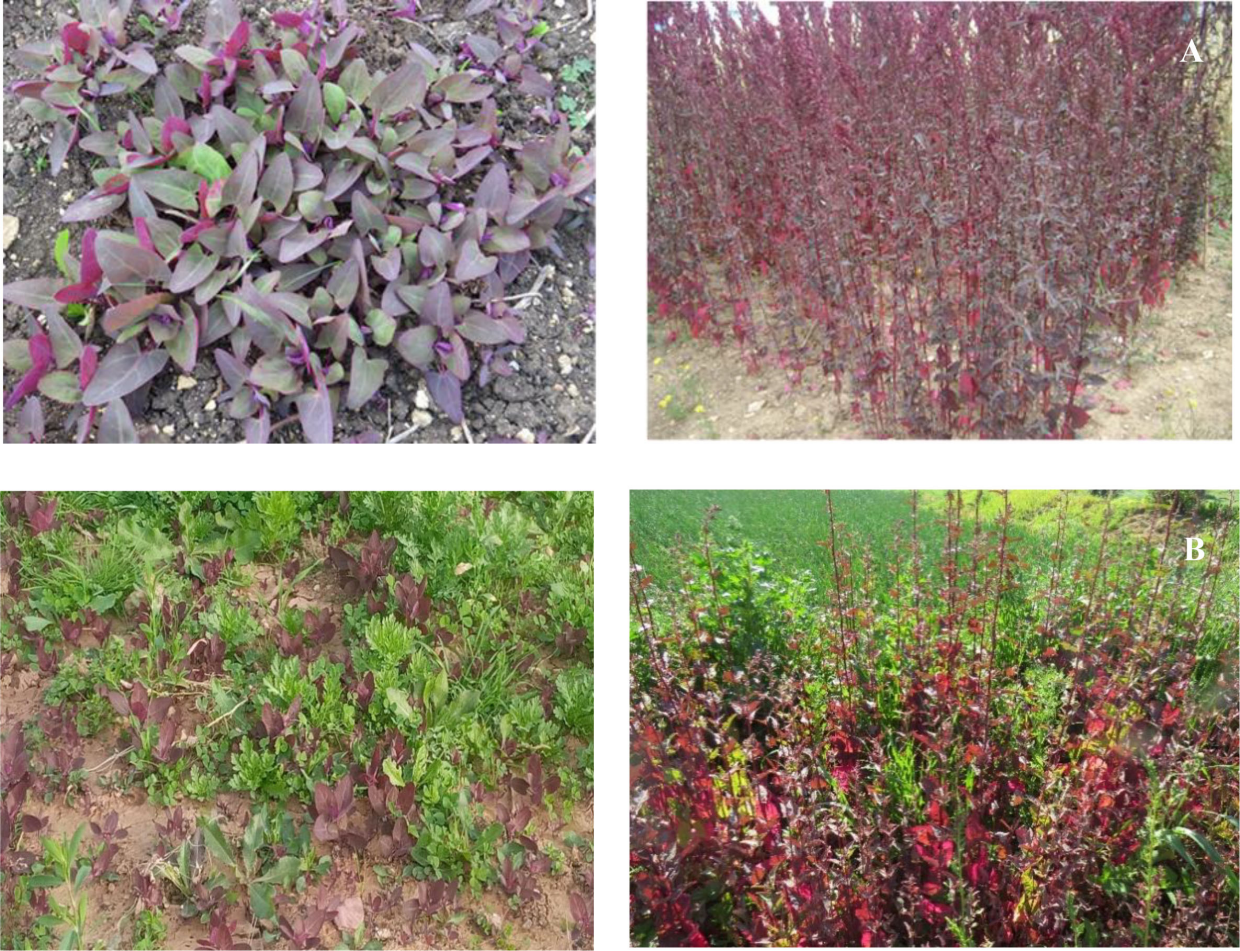
Growth of *Atriplex hortensis* under saline **(A)** and control **(B)** conditions at two critical development stages: early seedling establishment (30 days after sowing, DAS) and final vegetative stage (180 DAS).

To complement the growth data, representative field photographs of *Atriplex hortensis* under both control and saline conditions were captured at two critical developmental stages: early seedling establishment (30 DAS) and final vegetative growth (180 DAS) (**Figure 2**). These images highlight the plant’s ability to establish and maintain growth despite sustained salinity stress, while also illustrating the more vigorous development achieved under non-saline conditions.

### 3.2 Biomass partitioning and root-to-shoot ratios

**Figure 3:**
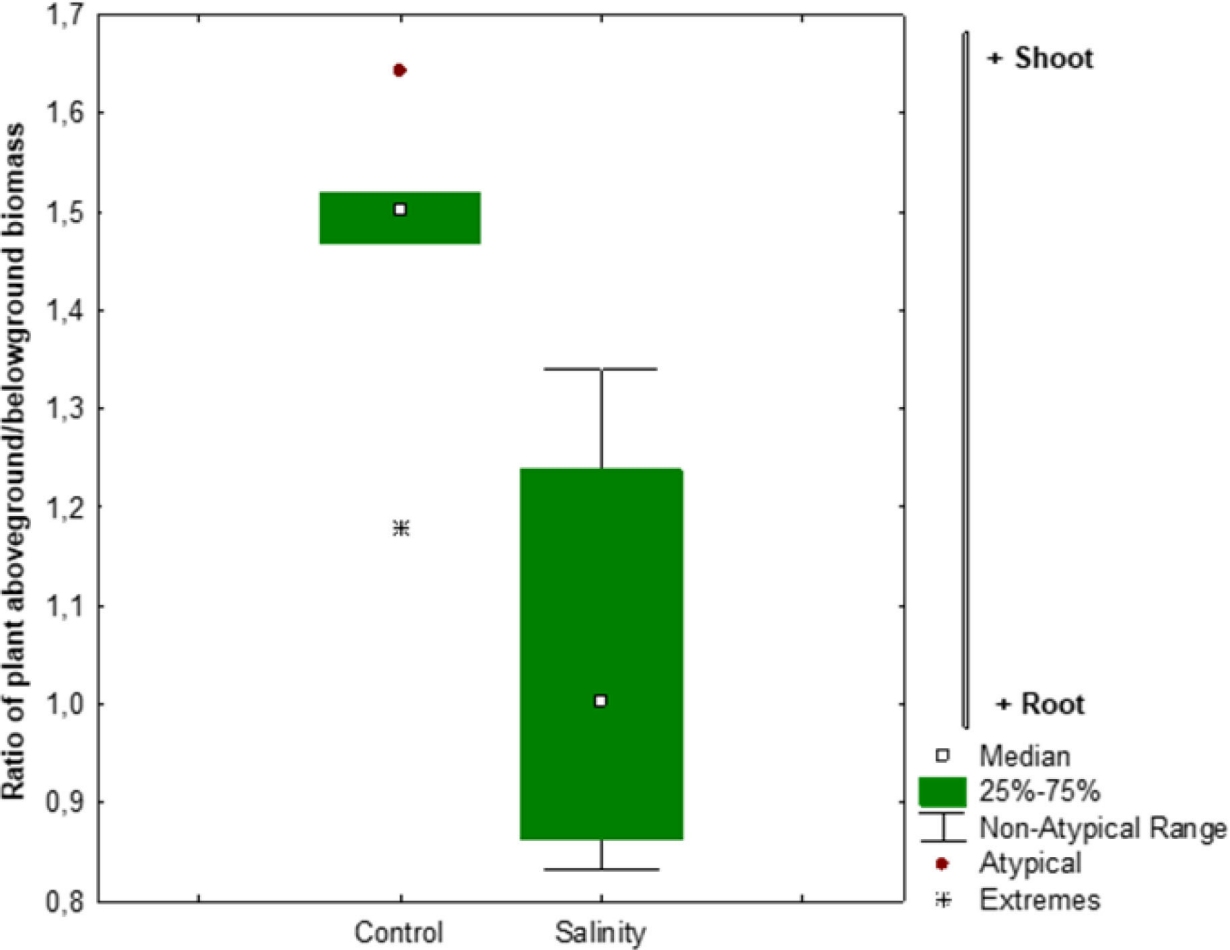
Response ratio of Atriplex plant aboveground: belowground biomass ratio in response to salinity stress. The error bars represent 95% confidence intervals. Asterisks indicate average response ratios as they differ from zero (P < 0.05).

Salinity stress significantly altered biomass partitioning in *Atriplex hortensis*, as shown in **Figure 3**. The shoot-to-root ratio decreased across all growth stages under saline conditions, indicating a strategic reallocation of resources. In control plants, shoots accounted for about 85% of total biomass, reflecting typical above-ground growth. Under salinity, this share dropped to approximately 75%, suggesting enhanced investment in root development. This shift likely reflects an adaptive response to improve water and nutrient uptake under osmotic stress. The consistent trend across developmental stages highlights the plant’s effort to strengthen below-ground functions critical for survival in saline environments, potentially compensating for reduced shoot expansion.

### 3.3 Effects of salinity on soil and plant parameters

Soil electrical conductivity (EC) in saline plots declined from 3.48 to 2.26 dS m^-1^ after six months of *Atriplex hortensis* cultivation, indicating a significant reduction in soil salinity. This decrease suggests effective salt uptake by the plants, confirming their role as biological desalination agents. Simultaneously, plant tissue EC increased from 3.5 to 8.4 dS m^-1^, reflecting active salt accumulation. This trait is typical of halophytes, which isolate salts through vacuolar sequestration, specialized salt bladders, and regulated ion transport. The concurrent reduction in soil EC and rise in plant tissue EC highlights *A. hortensis* as an efficient salt sink, contributing both to its own salt tolerance and to soil phytoremediation. These features make it a promising candidate for reclaiming salt-affected soils, particularly in regions where conventional methods are economically or logistically unfeasible.

Post-harvest analysis showed moderate improvements in soil chemical properties in both saline and control plots. The saline soil pH slightly decreased from 8.2 to approximately 7.9, while the control soil pH remained stable around 7.3. Exchangeable sodium levels in the saline plot reduced from 86 to about 70 mmol kg^-1^, suggesting sodium uptake by *Atriplex hortensis* and possible leaching, whereas the control plot remained nearly constant at 22 mmol kg^-1^. Potassium concentrations increased modestly in the saline soil from 34 to 40 mmol kg^-1^, while calcium and magnesium showed slight increases from 25 to 30 mmol kg^-1^ and 34 to 38 mmol kg^-1^, respectively, indicating partial recovery of nutrient availability and improved soil structure. Chloride content decreased from 118 to around 95 mg kg^-1^ in the saline plot, while remaining stable at 33 mg kg^-1^ in the control, further confirming a reduction in chloride-induced salinity stress. These changes reflect the positive impact of *Atriplex hortensis* cultivation in mitigating soil salinity and enhancing soil health under saline field conditions.

### 3.4 Leaf area and specific leaf area

**Figure 4:**
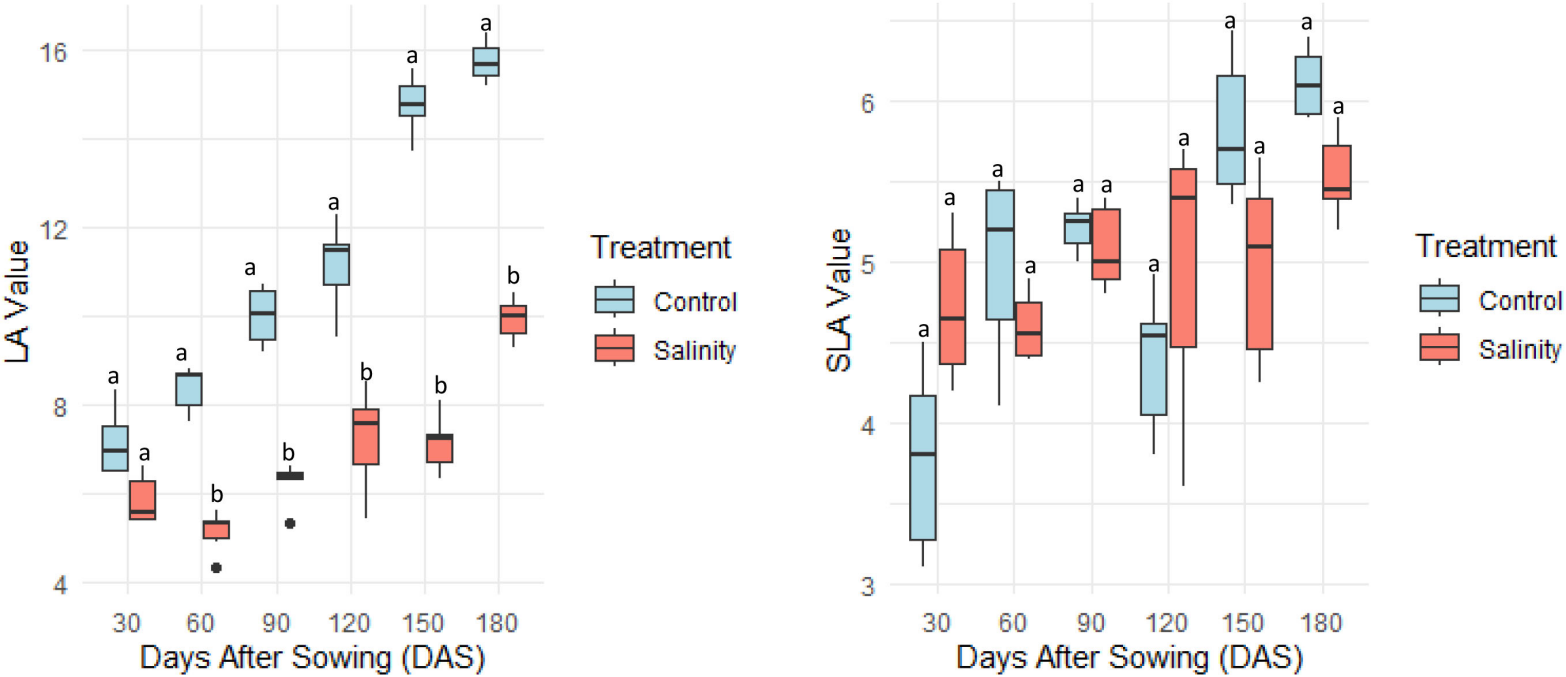
Leaf Area (LA, cm^2^) and Specific Leaf Area (SLA, cm^2^g^−1^) of *Atriplex hortensis* at successive growth stages under control and saline conditions (mean ± SD, n = 5). Different letters indicate statistically significant differences among groups (Tukey’s Honest Significant Difference, p < 0.05).

Leaf area (LA) increased progressively with plant development under both control and saline conditions; however, at each stage, LA was significantly lower under salinity, with an overall reduction of 35% compared to control plants at final stage (**Figure 4**). In contrast, specific leaf area (SLA) remained stable between treatments across all growth stages. The capacity to sustain SLA while moderating LA growth across stages illustrates a conservative strategy that optimizes water use efficiency and supports stress avoidance without compromising tissue functionality. This indicates that *Atriplex hortensis* maintained leaf tissue density and structural integrity despite salinity stress.

### 3.5 Physiological responses to salinity

**Figure 5:**
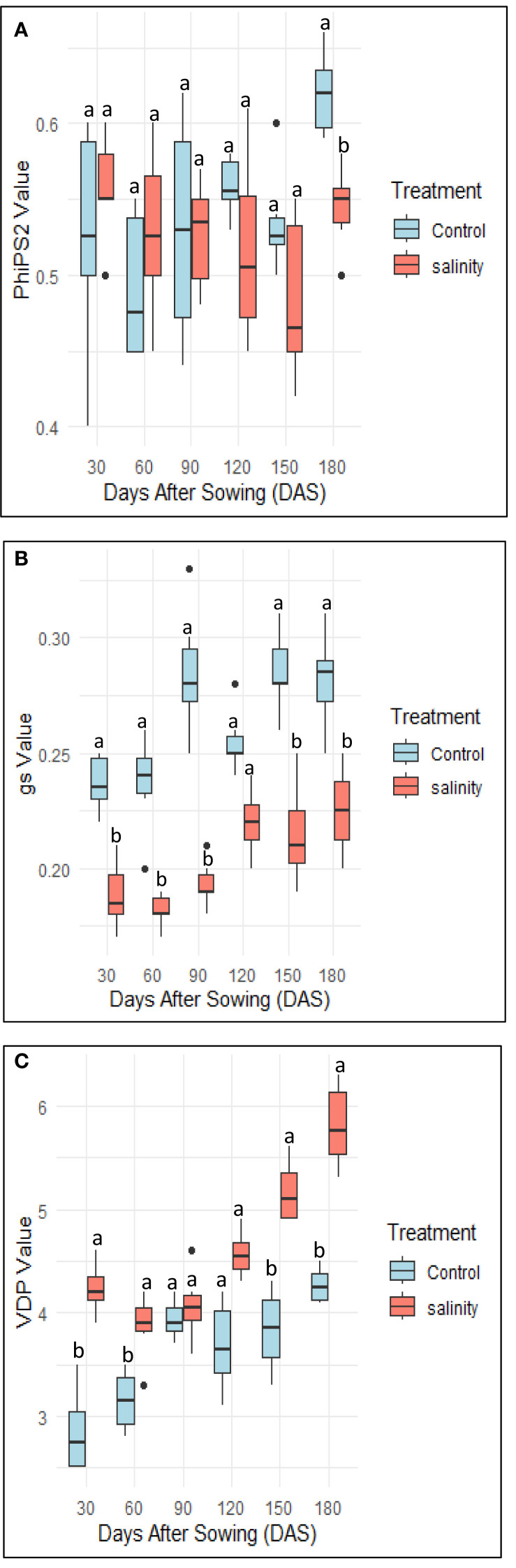
Boxplots comparing salinity stress response of the Atriplex plant (N = 10). Physiological measurements were taken with the LI-600 Porometer/Fluorometer (LI-COR Biosciences, Lincoln, NE, United States) and included **(A)** light-adapted chlorophyll fluorescence (PhiPS2 1-Fs/FMAX), **(B)** stomatal conductance (gs mol m^−2^ s^−1^) and **(C)** vapor pressure deficit (VPD kPa). Values are reported at successive growth stages under control and saline conditions. Different letters indicate statistically significant differences among groups (Tukey’s Honest Significant Difference, p < 0.05).

*A. hortensis* maintained stable midday leaf water potential (Ψ_h_) of approximately –3.2 MPa and high relative water content (RWC) above 88% across treatments, with no significant correlations observed. Salinity induced significant physiological stress in *Atriplex hortensis*, particularly from 120 days after sowing (DAS). Photosystem II efficiency (ΦPSII) declined significantly under salinity (−18.4%, *p* < 0.01), indicating reduced photochemical activity (**Figure 5A**). This was accompanied by a reduction in electron transport rate (ETR), reflecting impaired photosynthetic electron flow (**Table 3**). Stomatal conductance (*g*s) decreased progressively in saline-treated plants, stabilizing at 0.28 ± 0.02 mol m_-2_ s^-1^ compared to 0.45 ± 0.03 mol m_-2_ s^-1^ in controls (*p* < 0.001; **Table 3**, **Figure 5B**), suggesting a stomatal limitation to photosynthesis under osmotic stress. Vapor pressure deficit (VPD) increased steadily across growth stages under salinity, reaching 5.80 ± 0.08 kPa versus 4.27 ± 0.06 kPa in controls (*p* < 0.05) at 180 DAS (**Figure 5C**), indicating greater atmospheric water demand that likely exacerbated stomatal closure.

**Table 3:** Monthly dynamics of chlorophyll fluorescence in leaves of *Atriplex hortensis*.

Month	Fluorescence parameters					
	F0	Fm’	Fv/Fm	ETR	Gs	PSII
January	10.65 ± 1.2 ^bc^	27.49 ± 2.1^a^	10.67 ± 1.1^a^	0.3 ± 0.02^b^	0.20 ± 0.04^a^	0.4 ± 0.09^b^
February	9.32 ± 1.1^c^	23.7 ± 1.8^ab^	10.50 ± 1.2^a^	0.4 ± 0.06^b^	0.23 ± 0.07^a^	0.5 ± 0.07^ab^
March	14.16 ± 1.7^a^	21.9 ± 1.3^b^	10.57 ± 1.4^a^	0.5 ± 0.04^a^	0.18 ± 0.03^b^	0.6 ± 0.06^a^
April	13.24 ± 1.6 ^a^	22.1 ± 1.4^b^	10.92 ± 1.6^a^	0.6 ± 0.07^a^	0.21 ± 0.06^a^	0.5 ± 0.08^ab^
May	11.50 ± 1.4^b^	24.3 ± 1.7^a^	9.85 ± 1.3^b^	0.5 ± 0.05^a^	0.20 ± 0.07^a^	0.6 ± 0.05^a^
June	10.54 ± 1.2^bc^	23.5 ± 1.5^ab^	10.13 ± 1.3^b^	0.6 ± 0.05^a^	0.19 ± 0.04^b^	0.5 ± 0.04^ab^

Means not followed by the same upper case letter are different (P<0.05).

Mean values ± SE (n = 5–10). F0, fluorescence level; Fm, maximal fluorescence; Fv/Fm, maximum quantum efficiency of ФPSII; ETR, electron transport rates; gs, stomatal conductance; PSII, photosystem II photochemistry.

### 3.6 Heatmap analysis of morphological and physiological traits

**Figure 6:**
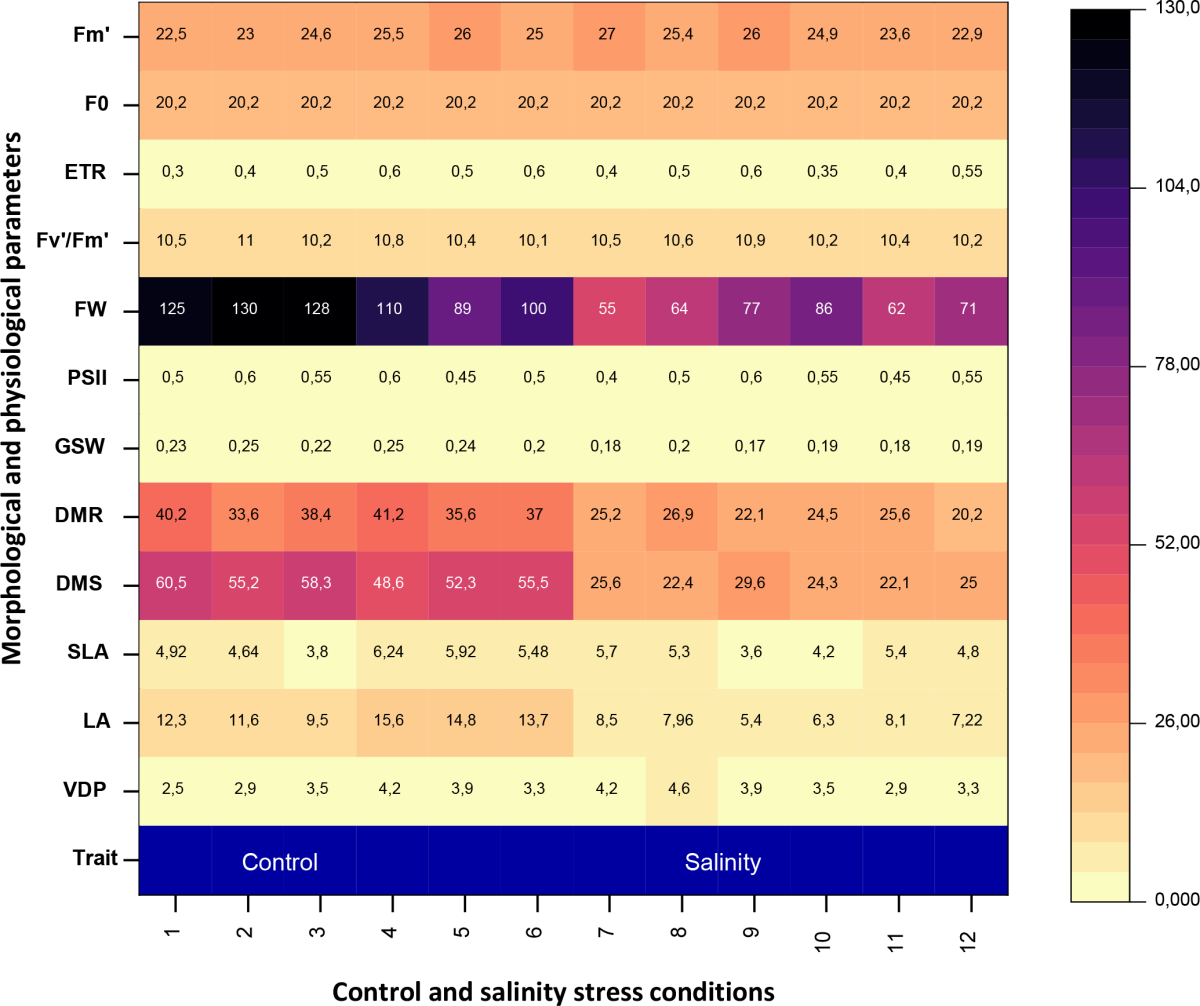
Heatmap for morphological and physiological parameters under control and salinity stress conditions in *Atriplex hortensis* plants after 6 months of treatment. VDP, vapor pressure deficit; LA, leaf area; SLA, specific leaf area; DMS, dry mass shoot; DMR, dry mass root; gs, stomatal conductance; PSII, photosystem II photochemistry; FW, fluorescence level; Fm’, maximal fluorescence; Fv’/Fm’ maximum quantum efficiency of ФPSII and ETR electron transport rates. Each column (x-values 1–12) represents an individual replicate: columns 1–6 correspond to control plants, and columns 7–12 to salinity-treated plants.

Heatmap-based hierarchical clustering clearly differentiated control and saline treatments based on their morphological and physiological responses to salinity (**Figure 6**). This analysis highlighted distinct clustering patterns, indicating consistent, stress-induced changes. Morphological traits particularly leaf area, shoot dry mass (DMS), and root dry mass (DMR) showed the greatest sensitivity to salinity, with marked reductions compared to physiological parameters.

In contrast, physiological traits such as relative water content (RWC) and leaf water potential (Ψ_h_) remained relatively stable across treatments, suggesting effective internal regulation. Notably, saline-treated plants exhibited the highest vapor pressure deficit (VPD) and the lowest photosystem II efficiency (ΦPSII), reflecting key adjustments to water loss and photochemical stress. Overall, the results demonstrate that *Atriplex hortensis* responds to salinity through both structural and functional changes, with morphological traits serving as more sensitive indicators of stress severity.

## 4 Discussion

The present study demonstrates that *Atriplex hortensis* can tolerate moderate soil salinity while contributing to soil remediation and sustaining key physiological functions. Although salinity stress reduced biomass production, the species exhibited adaptive strategies such as maintenance of relative water content and stable photosynthetic efficiency that enabled survival and growth over the six-month field trial. These findings are consistent with previous reports on the role of halophytes in restoring salt-affected soils (Qadir et al., 2007; Duarte and Caçador, 2021), and are further supported by recent studies highlighting the physiological plasticity and ion regulation mechanisms in halophytes under salinity and drought conditions (Ahmad et al., 2024; Ali et al., 2023). Moreover, the application of salt-tolerant species for phytoremediation has gained attention as an effective, low-cost approach for improving soil quality and supporting sustainable agriculture in degraded environments (Nguyen et al., 2024; Chourasiya et al., 2024; Chen et al., 2023).

### 4.1 Salt-induced growth inhibition and biomass allocation

Salinity significantly decreased both shoot and root biomass in *Atriplex hortensis*, consistent with earlier findings on *Atriplex* spp. that reported growth inhibition under saline conditions (Kachout et al., 2010; Wilson et al., 2000). The reduction in shoot biomass was more pronounced than that of roots, leading to a decreased shoot-to-root ratio under stress. This shift in carbon allocation toward root development is a typical adaptive response among halophytes, enhancing water uptake and nutrient acquisition in saline soils (James et al., 2006). Similar strategies have been observed in other salt-tolerant species such as *Salicornia europaea* and *Suaeda maritima*, where increased root growth supports salt exclusion and osmotic adjustment (Rozema and Schat, 2013; Alharby et al., 2023).

In forage legumes like *Medicago sativa*, salinity-induced root proliferation has also been associated with improved stress tolerance and nutrient foraging (Lazof and Bernstein, 1998). These responses are supported by recent research highlighting how halophytes maintain hydraulic conductivity and ion homeostasis to survive in high-salinity conditions (Flowers and Colmer, 2015; Bharti et al., 2021). In the present study, *A. hortensis* maintained relatively stable water potential and high relative water content under saline stress, indicating physiological resilience. Compared to other salt-tolerant forage crops, *A. hortensis* demonstrates a balanced trade-off between biomass reduction and stress adaptation. While total biomass declined, the plant preserved photosynthetic activity and accumulated moderate salt levels, confirming its potential for use as both a forage crop and a phytoremediation species in salt-affected agroecosystems (Chen et al., 2023; Mbarki et al., 2018; Nguyen et al., 2024).

### 4.2 Physiological stability under salinity stress

Despite reductions in stomatal conductance (gs), chlorophyll fluorescence (ΦPSII), and electron transport rate (ETR), *Atriplex hortensis* maintained high relative water content (RWC) and stable leaf water potential (Ψ_h_), indicating efficient osmotic adjustment and sustained cellular hydration key traits associated with salt tolerance (Munns and Tester, 2008). These physiological responses suggest that *A. hortensis* can regulate water loss while preserving tissue hydration, a characteristic observed in several halophytes capable of maintaining photosynthetic function under saline conditions (Bharti et al., 2021; Mbarki et al., 2018).

The observed increase in vapor pressure deficit (VPD) under salinity reflects greater evaporative demand, which is often linked to reduced transpiration. Elevated VPD has been shown to trigger stomatal closure as a protective mechanism against hydraulic dysfunction and excessive water loss (Grossiord et al., 2020). This coordinated reduction in gs, together with the maintenance of high RWC, aligns with a drought-avoidance strategy commonly reported in salt-tolerant plants (Ahmad et al., 2024; Chen et al., 2023). Such integrated physiological responses underscore the resilience of *A. hortensis* and support its suitability for cultivation in arid and salt-affected environments.

### 4.3 Leaf morphology and stress adaptation

The significant decline in leaf area (LA) under saline conditions, accompanied by a non-significant change in specific leaf area (SLA), suggests that *Atriplex hortensis* adopts a morphological strategy to reduce transpirational surface area while conserving leaf tissue density and functionality. This reduction in LA likely serves as a water-saving mechanism, limiting evaporative losses in response to osmotic stress, a response commonly observed in salt-tolerant species (Ahmad et al., 2024; Chourasiya et al., 2024). In contrast, the stability of SLA indicates that internal leaf architecture such as mesophyll thickness and chloroplast distribution remains largely unaltered, enabling the maintenance of photosynthetic performance per unit leaf mass (Bharti et al., 2021; Mbarki et al., 2018).

This trait conservatism in SLA under salinity may reflect the preservation of structural and biochemical components that support sustained carbon assimilation and metabolic function despite environmental stress. Moreover, conserving SLA under saline conditions may enhance long-term productivity and survival by minimizing the metabolic cost of anatomical remodeling. Such responses are consistent with recent findings in halophytes that emphasize the optimization of resource allocation between growth and stress tolerance mechanisms (Alharby et al., 2023; Nguyen et al., 2024).

### 4.4 Phytoremediation potential and soil salinity reduction

The observed reduction in soil electrical conductivity (EC) from 3.48 to 2.26 dS m^-1^ after six months of *Atriplex hortensis* cultivation, alongside a marked increase in plant tissue EC (from 3.5 to 8.4 dS m^-1^), underscores the species’ potential for phytodesalination. This notable shift in salt distribution from soil to plant biomass highlights the ability of *A. hortensis* to extract soluble salts from the rhizosphere and compartmentalize them within aerial tissues. Similar physiological mechanisms have been described in other halophytes, involving ion sequestration in vacuoles and epidermal structures, enabling continued growth under saline stress (Alharby et al., 2023; Ali et al., 2023). The higher pH and elevated exchangeable sodium levels in the saline soil are characteristic of sodic and alkaline soils, which negatively affect soil structure and fertility by displacing essential cations such as calcium and magnesium (Qadir et al., 2008). The observed reduction in Ca²^+^ and Mg²^+^ concentrations can impair nutrient availability and soil aggregation, leading to decreased plant growth (Aydi et al., 2008). Additionally, the high chloride content indicates chloride-type salinity, which increases osmotic stress on plants and can reduce water uptake (Munns and Tester, 2008). These challenging conditions highlight the relevance of halophytes like *Atriplex hortensis*, which possess mechanisms to tolerate and mitigate salt stress through selective ion uptake and compartmentalization (Ben Ahmed et al., 2008).

These findings reinforce the concept of biomass harvesting as an efficient means to remove salts from the soil system a core principle of phytodesalination (Nguyen et al., 2024; Ahmad et al., 2024). Biological salt extraction represents a cost-effective and environmentally sound approach for the remediation of salt-affected soils, particularly in arid and semi-arid environments where conventional chemical treatments may be unsustainable. Furthermore, the deployment of fast-growing, salt-tolerant species like *A. hortensis* aligns well with agroecological restoration strategies that combine productive forage cultivation with long-term land rehabilitation goals (Wang Y. et al., 2023; Wang S. et al., 2023).

### 4.5 Integration into saline agroecosystems

As an annual forage halophyte, *Atriplex hortensis* offers a dual advantage by combining biomass production with ecological restoration functions. Although total biomass is reduced under saline conditions, the yield remains sufficiently high to support its use as forage. The moderate salt accumulation in tissues makes it suitable for inclusion in mixed rations with salt-sensitive species, thereby diluting overall salt intake for livestock. Recent studies have highlighted the nutritional quality and safety of *A. hortensis*, demonstrating adequate crude protein levels and low anti-nutritional factors even under salinity stress, supporting its use in animal feed (Ali et al., 2023; Bharti et al., 2021).

Beyond its forage value, *A. hortensis* plays a vital role in soil stabilization and remediation. Its rapid canopy development provides ground cover that minimizes erosion, while its salt-extraction capacity contributes to reducing surface salinity via biomass harvesting. These traits make it particularly suitable for rehabilitating marginal or degraded lands. In vulnerable regions such as the Bizerte Lagoon, where seawater intrusion and inefficient irrigation exacerbate soil salinization, integrating salt-tolerant forage species like *A. hortensis* into land management can enhance ecosystem resilience and promote sustainable agriculture (Nguyen et al., 2024; Chourasiya et al., 2024).

*Atriplex hortensis*, like other halophytes, may engage in beneficial plant–microbe interactions that enhance its salt tolerance and phytoremediation potential, particularly through associations with rhizosphere bacteria and arbuscular mycorrhizal fungi (AMF) that improve nutrient uptake, alleviate ionic and oxidative stress, and contribute to soil structural and microbial restoration (Etesami and Beattie, 2018; Hashem et al., 2016; Szymańska et al., 2020). The feasibility of large-scale application of *Atriplex hortensis* in saline-affected landscapes relies on both agronomic performance and economic viability. Optimal planting density for forage halophytes generally ranges between 20,000 and 40,000 plants per hectare, allowing for efficient biomass production while minimizing resource inputs (Le Houérou, 2000). Although *Atriplex* species are recognized for their drought and salt tolerance, supplemental irrigation during the establishment phase can improve survival rates and early growth, particularly under arid and highly saline conditions (Glenn et al., 1999; Tlili et al., 2018). Once plant spacing and canopy structure are standardized, mechanized harvesting becomes feasible, reducing labor costs compared to manual harvesting typically used in traditional forage systems. Economic assessments indicate that halophyte-based systems offer a cost-effective strategy for rehabilitating marginal lands, particularly when their phytoremediation potential and sustained improvements in soil quality and productivity are considered (Qadir et al., 2008; Ventura et al., 2015). In this context, *A. hortensis* emerges as a valuable candidate for integration into saline farming systems, offering a dual benefit of forage biomass production and ecological restoration. Moreover, its use may contribute to carbon sequestration, enhance local biodiversity, and align with land restoration policies aimed at sustainable development and climate resilience in salt-affected Mediterranean regions.

## 5 Conclusion

This field study demonstrates that *Atriplex hortensis* exhibits notable physiological resilience to salinity stress, maintaining stable relative water content, leaf water potential, and stomatal conductance despite significant biomass reductions. Importantly, its cultivation under naturally saline conditions led to a measurable decline in soil electrical conductivity, confirming its phytodesalination capacity. These findings highlight *A. hortensis* as a promising candidate for integrated approaches to forage production and abiotic stress mitigation in salt-affected soils.

To advance its use under abiotic stress conditions, future studies should explore its long-term remediation potential, multi-season biomass productivity, and compatibility with salt-sensitive crops in intercropping systems. Investigating root-associated microbial communities, nutrient cycling dynamics, and economic feasibility under varying irrigation regimes will further support its adoption as a sustainable solution for managing salinity stress in arid and semi-arid agroecosystems.

## Data Availability

The original contributions presented in the study are included in the article/supplementary material. Further inquiries can be directed to the corresponding author.
